# The impact of rarity in NICE’s health technology appraisals

**DOI:** 10.1186/s13023-021-01845-x

**Published:** 2021-05-13

**Authors:** Sophie Clarke, Michelle Ellis, Jack Brownrigg

**Affiliations:** Pfizer Ltd, Walton Oaks, Dorking Road, Tadworth, Surrey KT20 7NS UK

**Keywords:** Orphan medicine, Rare disease, Population size, NICE, HST, STA, ICER thresholds

## Abstract

**Background:**

In the absence of a framework designed to evaluate medicines for rare diseases in the UK, most orphan medicines are appraised by the National Institute for Health and Care Excellence (NICE) through the Single Technology Appraisal (STA) process.

**Results:**

An analysis of STA appraisals of orphan and non-orphan medicines revealed that orphan medicines were subject to a significantly longer mean time in the NICE process than non-orphan medicines [370 days (n = 44) vs. 277 days (n = 118), *p* =  < 0.0001]. A higher proportion of orphan STAs required more than one Appraisal Committee Meeting (ACM) versus non-orphan STAs, and orphan STAs were disadvantaged by worse outcomes with respect to positive recommendations than those orphan medicines assessed by Highly Specialised Technology evaluation (HST).

**Conclusions:**

The uncertainties inherent to developing orphan medicines may contribute to these disadvantages. Improved understanding of the challenges in drug development for orphan medicines and clearer guidance for decision makers on navigating uncertainty in the HTA process may promote greater equity in access to medicines across rare and common conditions.

## Background

There are an estimated 6000–8000 rare diseases, and their cumulative impact is substantial. Over half are life limiting, three-quarters affect children and patients experience average delays of between six and eight years from the onset of symptoms to a definitive diagnosis [1, 2]. Diagnostic challenges are compounded by the lack of effective treatments as only around 5% of rare diseases have a licensed treatment [1–3].

There is no universally accepted definition of what constitutes a rare disease, however in the European Union (EU) any disease affecting fewer than 5 people in 10,000 is considered rare [4]. A subset of ultra-orphan diseases has been defined as those with a prevalence of ≤ 1 in 50,000 patients [5, 6]. This equates to approximately 28,143 patients for orphan conditions and 1126 patients for ultra-orphan conditions in England [7]. An orphan medicinal product is indicated for a rare disease and is required by the Committee for Orphan Medicinal Products to meet the following three requirements (1) indicated for a life threatening or serious condition affecting ≤ 5 in 10,000 people in the EU (2) unlikely that estimated sales would justify the Research and Development (R&D) investment, and (3) absence of other satisfactory treatment on the market for treating the rare disease [4, 8].

NICE evaluate medicines through a technology assessment process. A small subset of medicines for very rare conditions meet the strict criteria to follow the Highly Specialised Technology evaluation (HST) process with an Incremental Cost-effectiveness Ratio (ICER) threshold in excess of £100,000 per Quality Adjusted Life Year (QALY) gained [9, 10]. To meet HST criteria technologies must be licensed for a chronic and severely disabling condition; target a small distinct patient group; be expected to be used within a highly specialised service and concentrated in very few NHS centres; have the potential for lifelong use; be likely to have a high acquisition cost and the need for national commissioning is significant [11]. Most orphan medicines do not meet HST criteria and are evaluated through the standard NICE Health Technology Assessment (HTA) process, which is suited to the evaluation of treatments which are anticipated to provide benefit to large eligible populations. However there are concerns about the application of this to orphan medicines [9]. The standard ICER threshold under this process is £20–30,000 per QALY gained, and when assessed against these economic thresholds, orphan medicinal products often fail to meet the requirements due to high acquisition costs and a limited evidence base [9, 12]. To date, there has not been a specific QALY modifier for rarity [10].

We hypothesised that there is a subset of medicines for rare diseases, not evaluated under the HST framework or with an appropriate modifier in the STA process, which are subject to disadvantages. We conducted a systematic analysis of NICE appraisals between 2015 and 2020 to evaluate time in the NICE process and outcome stratified by population size or orphan designation.

## Methods

The analysis set of completed technology appraisals was downloaded from the NICE website and included those submitted between 01/01/2015 and 11/03/2020 to correspond with the first submission of HSTs in 2015. We further restricted our analyses to appraisals for “pharmaceutical” technology types. Where a product had been withdrawn from the market or there was no submission from the manufacturer, no data were extracted. Where an original appraisal had been replaced with a new appraisal, only the latest appraisal was included. For the extracted records, available data were collected from available NICE source documents and committee papers, accessed hierarchically, beginning with the most recent documents. We collected information on Technology Appraisal (TA) type, orphan designation (validated in EMA records), timeline of NICE appraisal process including appraisal committee meeting dates and final appraisal determination, NICE decision, number of patients eligible, incidence and prevalence of the condition.

Exclusion criteria included: appraisals with over 1000 days in the NICE process (these were individually assessed and excluded from time-based analyses if timelines could not be established), terminated appraisals and appraisals undergoing appeals.

Time in NICE was defined as submission to date of publication of final decision. NICE decisions were recorded as: Recommended (in line with label), Optimised/Restricted (for example recommended as second line option), Rejected (not recommended). The number of Appraisal Committee Meetings (ACMs) was calculated using the latest published dates for ACMs (e.g., if documents were published for ACM 3, but missing dates for ACM 1 & 2, it was assumed that there were 3 ACMs). Where data were recorded as ‘not found’, ‘not available’ or ‘redacted’ they were excluded from the analysis. Where a record was expressed as a range (e.g., number of eligible patients), the upper limit of the range was selected. Data on eligible population size were adjusted to represent England only for consistency. In cases where population sizes were expressed for the UK, or England and Wales, population data from the Office for National Statistics (ONS) mid 2019 report were used to derive adjusted estimates for England [7].

## Results

**Fig. 1:**
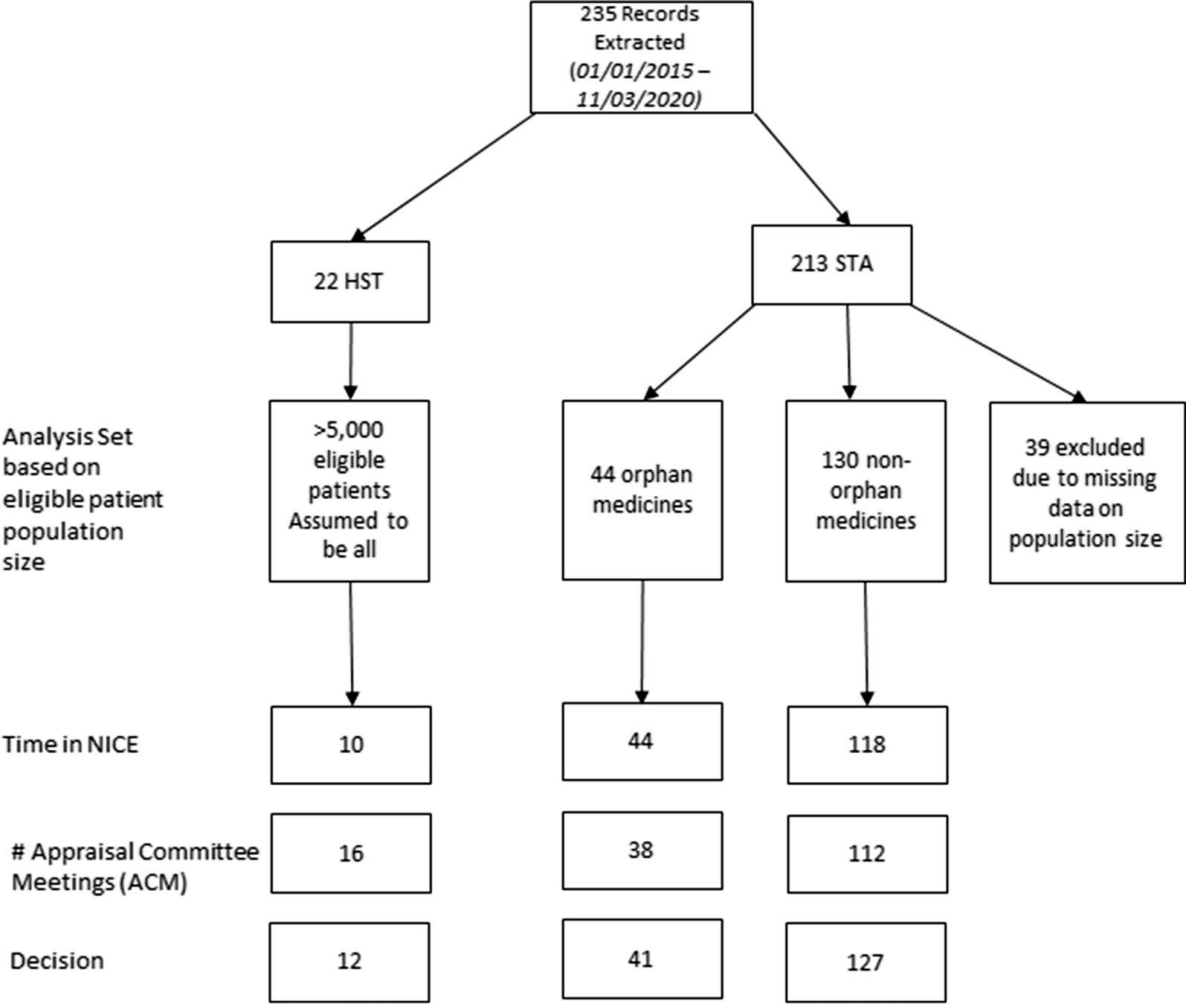
Prisma illustrating the analysis set of included STA and HST appraisals

235 records were extracted with submission dates between 01/01/2015 and 11/03/2020. Of these, 22 were HST appraisals (of which 10 were listed as in progress) and 213 STAs. Of the STA appraisals, 44 were listed as orphan medicinal products and 130 as non-orphan medicines. There were 39 STA appraisals with missing data on eligible population size which were excluded from the analyses (Fig. 1).

### Time based analyses

**Fig. 2:**
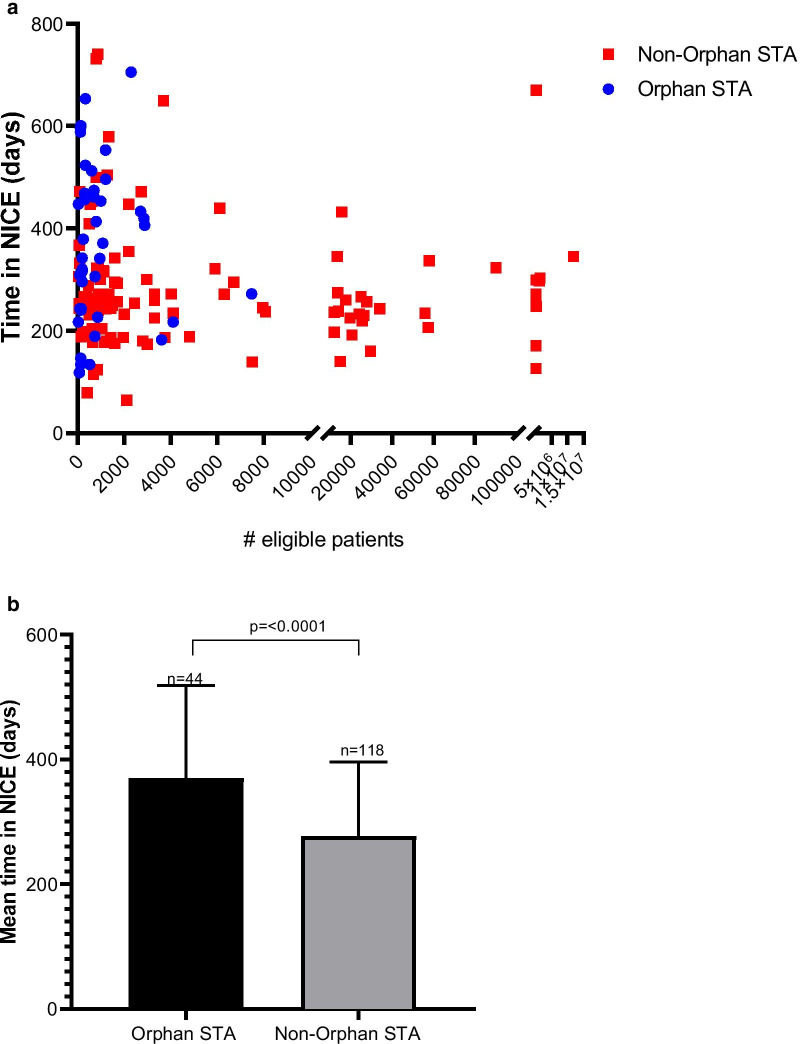
**a** Time in the NICE process by eligible population size and orphan status. **b** Mean time in NICE was significantly higher for orphan STA versus non orphan STA appraisals

Figure 2 illustrates that STA appraisals of orphan medicines had a significantly longer mean time in the NICE process than non-orphan medicines (370 days vs. 277 days, *p* < 0.0001). A further analysis by eligible population size demonstrated no significant difference in mean time in NICE for non-orphan STAs with an eligible population size < 5000 compared with > 5000 patients (284 vs. 270 days, *p* = 0.5). HST appraisals had a mean time in NICE of 397 days (n = 10) and there was no significant difference compared to orphan STAs (*p* = 0.6).

### Descriptive analyses

**Fig. 3:**
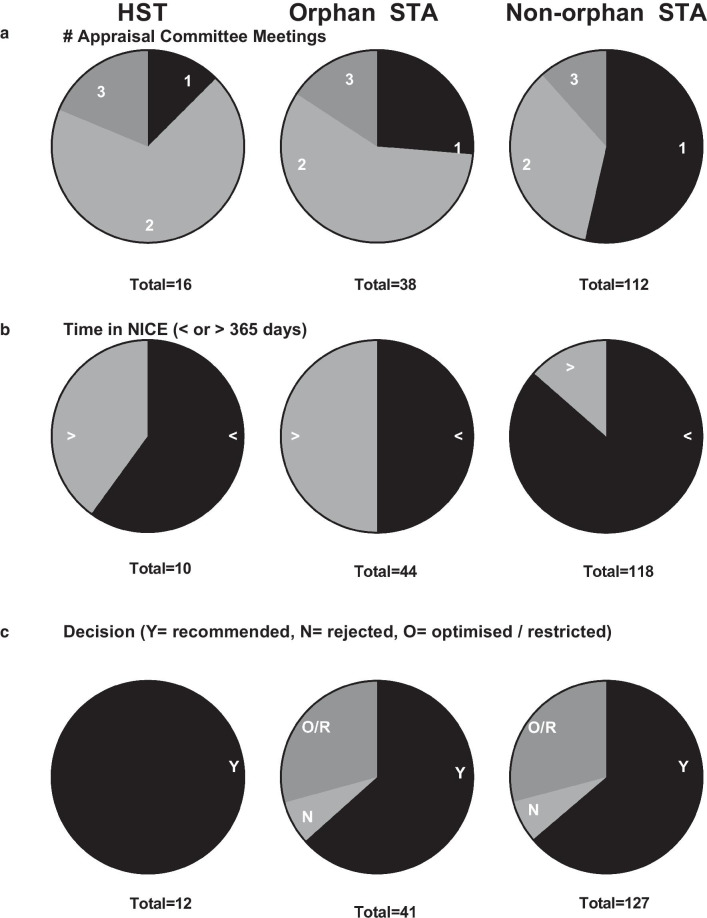
Descriptive analysis of HST, orphan STA and non-orphan STA appraisals. **a** Proportion of appraisal committee meetings, **b** proportion of appraisals with time in NICE < or > 365 days, **c** decision for completed appraisals

Figure 3 shows the number of ACMs, proportion of appraisals over 365 days in the NICE process, and the final decision for HST and STA appraisals. A higher proportion of both orphan STAs and HSTs required > 1 ACM compared to non-orphan STAs (74% and 88% vs. 46%, respectively). Non-orphan STAs had the lowest proportion of appraisals with a time in NICE > 365 days, while orphan STAs had the highest (14% non-orphan STA, 40% HST and 50% orphan STA). All HSTs were approved (n = 12), whereas the proportion of rejected, optimised or restricted outcomes for orphan STAs vs. non-orphan STAs were comparable (37% vs. 36%).

## Discussion

Orphan medicines undergoing STA appraisals are at a significant disadvantage in the current NICE process, facing higher numbers of ACMs and longer appraisal time than non-orphan STAs. The observed delays in reaching a decision to reimburse orphan medicines represent a “double-edged sword” for patients with rare diseases who often face a lengthy diagnostic odyssey lasting 6–8 years [2, 13].

Our analyses show the proportion of orphan medicines that receive a positive recommendation in the STA process is comparable with non-orphan medicines. This is somewhat surprising given that orphan medicines, by definition, address diseases without effective treatment options. Importantly, when compared to orphan medicines assessed through the dedicated appraisal framework for highly specialised medicines (HST), a higher proportion of orphan STAs took longer than a year (Fig. 3b) and were less likely to receive a ‘recommended’ outcome (Fig. 3c). It is acknowledged that ‘recommended’ outcomes may not be directly comparable between HST and STA appraisals. Under the HST process, allowances are made for the recommendation of technologies within conditional terms set by NICE including various requirements to collect additional data, stopping rules for therapy and further price negotiations as part of managed access arrangements. Under the STA process ‘recommended’ technologies may involve a patient access scheme, but only rarely further data collection, with the exception of drugs recommended for use under the Cancer Drugs Fund.

It should be noted that in our analysis, the number of absolute ‘rejections’ was higher for orphan STAs than for HSTs.

A previous report by Zamora et al. confirmed that a lower percentage of EMA approved orphan medicines are reimbursed in England compared with Spain, Germany, France and Italy [14]. Our findings suggest that this inequity in access to orphan medicines may be driven by challenges in the STA process. In addition, the Zamora analysis documented longer delays to reimbursement in England of over 2 years following EMA approval compared with 0–23 months across the four aforementioned countries. It should be noted that in their analysis most orphan medicines that were reimbursed in England were reimbursed because they were either included in the NHS England Specialised commissioning list or the Cancer Drugs Fund [14]. NICE positive decisions only accounted for a small proportion of the orphan medicines reimbursed [14].

From our analysis it was not possible to determine the reasons for the delays or reimbursement decisions. There are inherent difficulties in developing medicines for rare diseases including small population sizes in pivotal studies and consequently, limitations in the corresponding evidence package. It is recognised that orphan medicines often fail to meet the standard ICER threshold of £20–30,000 in the UK due to high acquisition costs and uncertainty in the evidence base [9, 10, 15].

In the absence of a uniform approach to addressing uncertainty in the STA process, clear guidance is needed for decision makers on how to allow for the uncertainties inherent to orphan medicines. It is crucial that this guidance is transparent for decision makers and reimbursement bodies so that manufacturers can address their needs in a forward manner in their clinical development programs.

There is an ongoing debate regarding the value that society place on rare diseases and what the corresponding provision in the NICE process should be [16]. The UK Strategy for Rare Diseases, recognises that in order for patients with rare diseases to have access to the most effective treatments, it is important to have appropriate, robust and transparent procedures which should be able to take account of the particular challenges associated with evaluating treatments for rare diseases [17]. This is supported by the recently published UK Rare Disease Framework which highlights the importance of improving access to specialist care, treatments and drugs [13].

The ongoing NICE Methods Review (NMR), initiated in 2020, aims to ensure that the NICE methods are robust and up to date [18]. Current proposals in the NMR include a revision of modifiers, to update the current modifier for life extending treatments with a new modifier for severity of disease. Beyond this, there is an acknowledgement in the proposals that there should be a greater degree of acceptance of uncertainty and risk in defined circumstances, such as rare diseases, innovative technologies, and technologies with significant benefits. The importance of managed access agreements in monitoring and controlling this uncertainty and risk is also emphasised. A modifier to address health inequalities has also been proposed and although details remain unclear, we suggest that this could help to address inequity of access to orphan medicines. These proposals are encouraging in light of our findings; however, it remains to be seen whether more specific guidance will be provided to decision makers.

### Limitations

We acknowledge our analyses are subject to possible bias introduced through the assumptions made to standardise the data for analysis: (1) where data were expressed as a range, we selected the upper limit, (2) where not all dates were available for ACMs, we took the last available date as the final meeting, however it is possible that there were further meetings without published papers. (3) data on eligible population size was adjusted to represent England only for consistency, and published data was assumed to represent England and Wales if not specified, (4) there were a large number of non-orphan STA appraisals with eligible population sizes quoted in NICE documents < 5000 and this may be a reflection of inconsistency in the way eligible population size is calculated and submitted to NICE by manufacturers or reported in the NICE documents. Furthermore, we excluded 39 STA appraisals due to missing data on eligible population size. Finally, the scope of this analysis did not cover technologies for rare conditions which are assessed by the Coalition of Patient Advocacy Groups (CPAG) and do not undergo NICE appraisal or allow for the evaluation of any potential special conditions associated with HST approvals. There was insufficient data to allow evaluation of the impact of the cost of medicines on the NICE process or outcomes. Terminated/withdrawn appraisals were not included, and these may have included appraisals for orphan medicines which were withdrawn at the decision of the manufacturer due to inability to meet the standard STA ICER threshold. The disadvantage faced by orphan medicines undergoing STA appraisals could therefore be underestimated in this analysis. We believe that further evaluation of these appraisals is important.

It's important to recognise that not all orphan medicines are subjected to the same challenges. Whilst the scope of our analysis did not allow for separation of oncology orphan medicines, there are indicators that these may be less disadvantaged than other orphan medicines. For example, they have been shown to have comparable evidence standards to non-orphan oncology products, and HTA decisions for oncology medicines can be influenced if they meet NICE criteria for End-of-Life [15]. Further analysis to identify the group of orphan medicines remaining at a disadvantage under the current NICE methods would be worthwhile to inform the NMR so that adequate provision can be made to ensure equity of access for all patients with rare conditions.

## Conclusion

In the absence of an appropriate modifier within the STA process or an appraisal framework designed to evaluate therapies for rare diseases, orphan products are subject to unwarranted disadvantages in the NICE process with respect to both the time taken to reach a decision and the proportion of recommended outcomes. By definition orphan products address unmet therapeutic needs for patients with rare conditions. It is critical that access to these therapies is not precluded by uncertainties that are unavoidable in the context of the diseases they treat. Improved understanding of the challenges in drug development for orphan medicines and clearer guidance for decision makers on navigating uncertainty in the HTA process may promote greater equity in access to medicines across rare and common conditions.

## Data Availability

The datasets used and/or analysed during the current study are available from the corresponding author on reasonable request.

## Abbreviations

ACM: Appraisal committee meeting
CPAG: Coalition of Patient Advocacy Groups
EMA: European Medicines Agency
EU: European Union
HTA: Health technology appraisal
HST: Highly specialised technology evaluation
ICER: Incremental cost-effectiveness ratio
NICE: National Institute for Health and Care Excellence
NMR: Nice methods review
ONS: Office for National Statistics
QALY: Quality adjusted life year
R&D: Research and development
STA: Single technology appraisal
TA: Technology appraisal
